# Bat-Borne Coronaviruses in Jordan and Saudi Arabia: A Threat to Public Health?

**DOI:** 10.3390/v12121413

**Published:** 2020-12-09

**Authors:** Laith N. AL-Eitan, Amneh H. Tarkhan, Mansour A. Alghamdi, Denise A. Marston, Guanghui Wu, Lorraine M. McElhinney, Ian H. Brown, Anthony R. Fooks

**Affiliations:** 1 Department of Biotechnology and Genetic Engineering, Jordan University of Science and Technology, Irbid 22110, Jordan; amneht92@gmail.com; 2 Department of Anatomy, College of Medicine, King Khalid University, Abha 61421, Saudi Arabia; m.alghamdi@kku.edu.sa; 3 Genomics and Personalized Medicine Unit, College of Medicine, King Khalid University, Abha 61421, Saudi Arabia; 4 Department of Virology, Animal and Plant Health Agency (APHA, Weybridge), Surrey KT15 3NB, UK; Denise.Marston@apha.gov.uk (D.A.M.); guanghui.wu@apha.gov.uk (G.W.); Lorraine.McElhinney@apha.gov.uk (L.M.M.); Ian.Brown@apha.gov.uk (I.H.B.); Tony.Fooks@apha.gov.uk (A.R.F.)

**Keywords:** bats, coronaviruses, emerging infectious disease, viral disease, zoonotic disease, COVID-19, Jordan, Saudi Arabia

## Abstract

Emerging infectious diseases are of great concern to public health, as highlighted by the ongoing coronavirus disease 2019 (COVID-19) pandemic. Such diseases are of particular danger during mass gathering and mass influx events, as large crowds of people in close proximity to each other creates optimal opportunities for disease transmission. The Hashemite Kingdom of Jordan and the Kingdom of Saudi Arabia are two countries that have witnessed mass gatherings due to the arrival of Syrian refugees and the annual Hajj season. The mass migration of people not only brings exotic diseases to these regions but also brings new diseases back to their own countries, e.g., the outbreak of MERS in South Korea. Many emerging pathogens originate in bats, and more than 30 bat species have been identified in these two countries. Some of those bat species are known to carry viruses that cause deadly diseases in other parts of the world, such as the rabies virus and coronaviruses. However, little is known about bats and the pathogens they carry in Jordan and Saudi Arabia. Here, the importance of enhanced surveillance of bat-borne infections in Jordan and Saudi Arabia is emphasized, promoting the awareness of bat-borne diseases among the general public and building up infrastructure and capability to fill the gaps in public health preparedness to prevent future pandemics.

## 1. Introduction

Experts have been warning about the possibility of a pandemic threat, and the lack of preparedness at national levels, for many years [1]. Despite such warnings and in spite of the existence of the World Health Organization (WHO) International Health Regulations (IHR), coronavirus disease 2019 (COVID-19) still caught the majority of the world off-guard, and some governments struggled to contain the viral outbreak as it spread through populations worldwide. Healthcare systems in many countries have been overwhelmed as the spread of COVID-19 resulted in a shortage of healthcare workers and resources [2]. The COVID-19 pandemic is a stark reminder of the ongoing global public health challenges created by emerging infectious diseases and global movements of people and animals, prompting the need for “robust research to understand the basic biology of new organisms and our susceptibilities to them” [3].

Emerging infectious diseases, which are diseases that have appeared or affected a population for the first time or have existed previously but are rapidly increasing either by the number of cases or by geographical spread, pose a major threat to human health and are often of zoonotic origin [4]. The emergence of infectious disease is partly driven by environmental changes that affect interactions between humans and animals in such a way so as to initiate a cross-species transmission (CST) event, or a host switching event [5]. CST occurs when a virus spreads to a new host species in a sustained manner after initial infection, i.e., successfully completes the viral infectious cycle in the new host species [6]. This may or may not lead to onward transmission in the new host species. If adaptation of the virus occurs resulting in sustained onward transmission, it is a host switching event. In many cases, infectious disease emergence in humans arises via an amplifier and intermediate hosts, exposing humans to pathogens from animals that would normally have little human contact [7]. Some examples of emerging infectious diseases that spread to humans through intermediate hosts include human coronavirus 229E (HCoV-229E), which is transmitted to humans through camelids, and severe acute respiratory syndrome coronavirus (SARS-CoV), which is transmitted to humans through palm civets [8].

Bats have been identified as natural reservoirs for many families of viruses that cause clinically relevant diseases in humans, including bunyaviruses (febrile diseases), coronaviruses (mild and severe respiratory tract infections), hepaciviruses (hepatitis), herpesviruses (skin lesions and malignancies), lyssaviruses (rabies), orthoreoviruses (mild gastroenteritis and upper respiratory infection), paramyxoviruses (measles and mumps), and pegiviruses (encephalitis), among others [9,10,11]. New analyses reveal that the number of zoonotic viruses in avian and mammalian orders increases in proportion to the number of species, and, as a diverse mammalian order themselves, bats could potentially carry many zoonotic viruses [12].

Due to frequent CST events, bat-borne viral infections are a major cause of emerging infectious diseases in humans via a number of transmission mechanisms, which include bat bites and scratches, exposure to bat urine, and ingestion of bats [13]. Particularly, viral shedding, which is the release of viruses from host cells, in bats has been reported to coincide with the peak periods of CST to human and other animal populations [14,15,16,17]. Some of the most prominent host-switching events to humans have involved coronaviruses, resulting in the zoonotic introduction of severe acute respiratory syndrome (SARS)-CoV, Middle East respiratory syndrome (MERS)-CoV, and, most recently, SARS-CoV-2, which is responsible for the ongoing pandemic.

The Eastern Mediterranean and Southeast Asian regions are highly vulnerable to emerging infectious and parasitic diseases, which are responsible for around 15% of morbidities and mortalities in the Eastern Mediterranean [18]. In fact, pneumonia and acute respiratory infections were reported to be a leading cause of death in the Eastern Mediterranean region, and they were often associated with zoonotic pathogens such as avian influenza A, the pandemic H1N1/09 virus, and MERS-CoV [19]. Alarmingly, the public healthcare systems in almost every Eastern Mediterranean country were inadequately prepared to respond effectively to a viral epidemic [20]. This inadequacy has only been exacerbated by the ongoing wars, civil unrest, population growth, and an aging population, which have all contributed to the spread of communicable diseases in a region which is already regularly exposed to mass influx events and mass gatherings [21].

The Arab world, which is a part of the Eastern Mediterranean region, is no stranger to the challenges posed by mass influx events and mass gatherings, as can be clearly seen in the cases of Jordan, with its role in the Syrian refugee crisis, and Saudi Arabia, as the main destination of religious pilgrimage. In fact, the annual mass religious gatherings that occur in Eastern Mediterranean countries such as Iran, Iraq, and Saudi Arabia have been cited as an additional challenge that complicates SARS-CoV-2 containment efforts in the region [22]. Additionally, refugee populations have been identified as having a heightened level of vulnerability to SARS-CoV-2, as their living conditions can make it difficult to practice the necessary public health measures, including physical distancing, self-isolation, and quarantine [23]. As a result, the present review was carried out to explore the public health preparedness in Jordan and Saudi Arabia for future pandemics, the possibility of which is exacerbated by the presence of native bat species whose habitats are increasingly being encroached upon by human populations.

## 2. Mass Influxes and Gatherings

Infectious diseases pose a significant risk to mass gathering and influx events, as they can easily spread between individuals and overwhelm the host country’s healthcare system [24]. Mass influxes occur when a large number of displaced individuals from a particular country arrive in a community [25]. Similarly, a mass gathering can typically be defined as the presence of at least 1000 people at a particular location for a specific amount of time, but it can also involve events that are attended by enough people to strain the resources of the host community in which they are held [26].

### 2.1. Jordan

Throughout its history, Jordan has experienced several mass influxes of displaced persons and refugees from Palestine, Lebanon, Kuwait, Iraq, and, most recently, Syria [27]. The Syrian Civil War has forced more than 1.2 million Syrians to flee to neighboring Jordan, but just over 620,000 are legally registered as refugees [28,29]. The Zaatari refugee camp, which is located just 12 km from the Jordan-Syria border, has become the fourth largest Jordanian city in terms of the population [30]. However, the majority of Syrian refugees do not live in refugee camps, instead choosing to reside in rural communities in the northern governorates of Irbid and Mafraq as well as in urban settings in the capital of Amman [31]. Syrian refugees were initially allowed access to free healthcare in Jordanian public hospitals, but the increasing burden on the public healthcare system resulted in revised policies that required Syrian refugees to pay unsubsidized healthcare rates [32,33]. In 2019, the Jordanian government reversed its health policy towards Syrian refugees, granting them access to subsidized healthcare once again [34].

In recent years, the public health epidemiological profile of refugee populations has shifted away from communicable diseases, as illustrated by the increasing non-communicable disease burden suffered by Syrian refugees in Jordan [35]. Among Syrian refugees, non-communicable diseases such as cancer have constrained the tertiary healthcare sector in Jordan [36]. Although no major infectious disease epidemic has occurred in Jordan, an increasing number of outbreaks have appeared among both the Jordanian and Syrian communities [37]. Cases of hepatitis A, leishmaniasis, and tuberculosis have occurred frequently in refugee camps, and polio outbreaks in neighboring countries have put Jordan at increased risk, which was mitigated with a nation-wide immunization program [38,39,40].

### 2.2. Saudi Arabia

Each year, millions of people from around the world arrive in the city of Mecca to perform either the greater pilgrimage (Hajj), which is performed at a certain time of the lunar year or the lesser pilgrimage (Umrah), which can be performed on a year-round basis [41]. Both types of pilgrimages involve mass gathering events, but the number of Hajj pilgrims far surpasses the number of Umrah pilgrims at any given time, as the five-day Hajj season causes the population of Mecca to triple as more than 2 million pilgrims descend upon the city [42]. Although the Umrah can be completed in a few hours, international pilgrims often take advantage of the two-week Umrah visa to visit other holy sites within the country [43].

A combination of the hot desert climate, physical fatigue, and crowded conditions makes pilgrims much more susceptible to contracting an infectious disease, especially acute respiratory infections [44]. Moreover, the fact that the Hajj is based on a lunar calendar means that it moves forward by 10 to 11 days each year, causing extra challenges based on whether it coincides in the hot summer months or during influenza season in the Northern hemisphere [42]. Increasing global temperatures due to climate change are only expected to exacerbate heat-related illnesses, including heat-stroke and heat exhaustion, among Hajj pilgrims [45].

From a historical perspective, the Hajj has been affected by a number of outbreaks involving various meningococcal diseases in 1987, 2000, and 2001 [46]. In addition, gastroenteritis and diarrhea feature prominently among pilgrims, with rates of prevalence ranging between 2% and 23% [47]. During the 2011 to 2013 Hajj seasons, a study of fecal samples from pilgrims suffering from diarrheal illness found that bacteria were the most common foodborne pathogens, comprising *Salmonella* spp., *Shigella*, and *E. coli* [48]. Among pilgrims returning from the Hajj, increased acquisition rates of multi-drug resistant *Salmonella* spp., *E. coli*, and *A. baumannii* were observed [49,50].

## 3. Distribution of Bats and Associated Pathogens

Bats belong to the diverse order Chiroptera, comprising over 1300 species that can be found on every continent except Antarctica [51]. Although they play an integral role in pest control, pollination, and seed dispersal, bats are hosts to a diverse number of viruses with high zoonotic potential and frequent spill over into human populations [52,53,54]. In fact, bats are known to be the natural reservoirs of SARS-like coronaviruses, with three *Rhinolophus* species (*R. macrotis*, *R. pearsoni*, and *R. pussilus*) demonstrating a high prevalence of SARS-CoV antibodies [55]. It is hypothesized that bats act as major viral reservoirs due to dampened inflammation, which allows viral infections to persist asymptomatically, high interferon levels, which limit viral replication, and a highly similar major histocompatibility complex class II (MHC II) human leukocyte antigen DR isotype (HLA-DR), which facilitates cross-species zoonotic infection [56,57].

**Figure 1:**
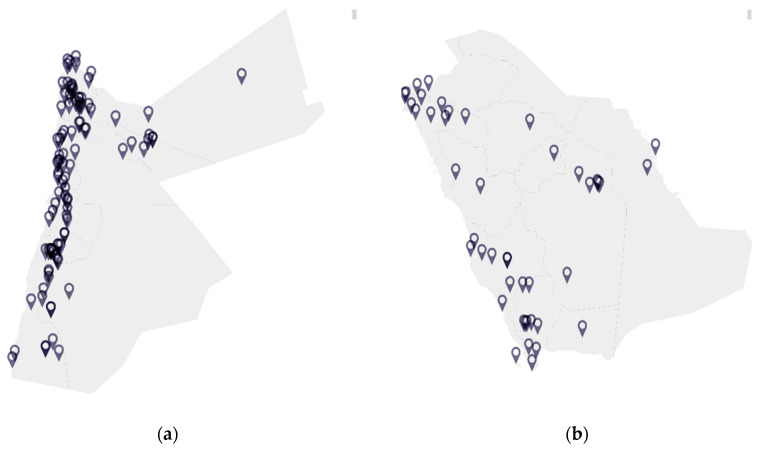
Distribution of bat species in (**a**) Jordan and (**b**) Saudi Arabia.

A dearth of information exists in the context of viral surveillance of bats in Jordan, Saudi Arabia, and the wider Eastern Mediterranean region [58]. Figure 1 illustrates the distribution of bat species that have been reported in Jordan and Saudi Arabia based on the published literature, while Table 1 lists the species of bats found in Jordan and Saudi Arabia along with their associated viral pathogens worldwide. *Pipistrellus kuhli*, which is found in both Jordan and Saudi Arabia, is of particular interest as a reservoir for a number of viral pathogens, resides in urban areas, and often comes into close contact with humans [59]. *Rousettus aegyptiacus* is also resident in Jordan and Saudi Arabia and has been previously been reported to be infected with the Lleida bat lyssavirus [60].

### 3.1. Jordan

Bats constitute the largest diversity of mammalian species in Jordan, comprising at least 24 species from 8 families representing almost half of all bat species recorded in the Middle East and Egypt [61]. There is very little information about the pathogens harbored by Jordanian bats. In Europe, *Eptesicus serotinus* is responsible for human and animal exposure to the European bat 1 lyssavirus (EBLV-1) [62,63]. Other bat species known to carry lyssaviruses are *Miniopterus schreibersii* (a reservoir that sustains EBLV-1 transmission in multispecies bat populations), *Myotis capaccinii* (a regional migrant), and *Rhinolophus ferrumequinum* are also found in Jordan [64,65]. Coronaviruses have also been associated with several bat species native to Jordan (Table 1).

### 3.2. Saudi Arabia

There is a dearth of research on the bats of Saudi Arabia, and bats are considered to be a rare sight in the country, particularly in its central desert region [66]. Nonetheless, at least 15 species of bats from 8 families have been recorded in Saudi Arabia, including Mecca, Medina, Jeddah, and Riyadh [66]. MERS-CoV has been isolated from *Rhinopoma hardwickii* as well as *Taphozous perforatus*, and a number of other species native to Saudi Arabia have been previously associated with a range of coronaviruses as well as EBLV-1 (Table 1).

Although they can be sources of viral diseases, little is known about bats and the extent of their contact with human populations in Jordan and Saudi Arabia. It has been observed that bat guano, the excrement of bats, is used as a source of natural fertilizer by farmers in a local context, as evidenced by the collection of guano from caves in Northern Jordan [101]. Consequently, future work should continue to investigate the molecular epidemiology of different virus isolates to improve our understanding of zoonotic viral diseases in humans and animals.

## 4. Coronaviruses in the Middle East: MERS-CoV and SARS-CoV-2

First discovered in the 1960s, the coronaviruses (CoV) are a group of enveloped single-stranded RNA viruses with a large genome that can infect different species and cell types [102,103]. Within the past few decades, CoVs have been thrust into the media spotlight after being identified as the cause of a number of lethal diseases in humans. In fact, SARS-CoV-2 is the seventh CoV that has been identified to cause known disease in a human host [104]. CoVs were not reported as serious public health concerns prior to 2003, as their pathogenesis was limited to mild upper respiratory infections [105]. In fact, four human CoV (HCoV-229E, HCoV-HKU1, HCoV-OC43, and HCoV-NL63) are believed to contribute to approximately 30% of common cold cases in the global population [106]. However, in 2002, SARS-CoV was introduced into the human population, followed by multiple zoonotic introductions of MERS-CoV in 2012 [107,108].

The origin of MERS-CoV is the subject of conflicting findings. Several studies have identified a high viral load of MERS-CoV in different bat species, while others suggest that MERS-CoV could not have been transmitted to humans by bats [109,110,111]. Despite this, the use of anti-MERS-CoV antibodies revealed that 98–100% of studied camels in different Middle-Eastern countries were positive for MERS-CoV [111]. Correspondingly, the prevalence of MERS-CoV in humans was found to be 15 times higher for camel shepherds and 23 times higher for slaughterhouse workers than for the general population [111]. On a similar note, SARS-CoV-2 emerged in late 2019 and is posited to have been introduced into the human population from bats (possibly *Rhinolophus* spp.); however, its potential intermediate host, if any, remains undetermined [112]. Some bat species are a natural reservoir for SARS-like CoVs, several of which have shown a serious potential to infect humans [55,113,114,115].

In terms of the case fatality rate (CFR), it appears that MERS-CoV is more deadly than SARS-CoV-2. MERS-CoV has a CFR of 34.4%, while, as of 18 November 2020, SARS-CoV is estimated to have a CFR of 2.4% [116,117]. With regard to transmission, SARS-CoV-2 is generally more transmissible than MERS-CoV, with the former estimated to have an overall reproductive number (R0) of 4.5 compared to the latter’s overall R0 of 0.45 in Saudi Arabia [117,118,119]. However, it must be noted that the extent of testing ramp-up as well as the role of ’super-spreaders’, i.e. individuals who have a "propensity to infect a larger than average number of susceptible individuals", can impact the R0 across different regions [119].

### 4.1. Middle East Respiratory Syndrome-Related Coronavirus (MERS-CoV)

MERS is also known as “camel flu” and is caused by infection with the MERS-related coronavirus (MERS-CoV), resulting in coughing, diarrhea, dyspnea, and fever in humans [120]. Symptoms in humans can be mild, but, at its most severe, MERS can result in highly lethal pneumonia as well as renal dysfunction and failure, especially in individuals with pre-existing conditions [121]. MERS-CoV is endemic in the dromedary camels (*Camelus dromedarius*) of the Arab region, with an increasing number of phylogenetic and surveillance studies pointing towards a bat origin of the virus [122,123]. Despite a number of outbreaks in recent years, the overall risk posed by MERS-CoV to the global population is somewhat low in terms of its pandemic potential, and the virus has not yet fully evolved for efficient human-to-human transmission [124].

MERS-CoV was first isolated in Jeddah, Saudi Arabia, from a 60-year old male presenting with acute respiratory symptoms that culminated in his death from progressive respiratory and renal failure [67]. The African *Neoromicia* bats have been posited to be natural reservoirs of the MERS-CoV, and a MERS-CoV isolate from an Egyptian tomb bat (*Taphozous perforatus*) was shown to be 100% identical to an isolate from the index case in the 2012 outbreak [125]. In experimental studies, MERS-CoV has been shown to replicate in Jamaican fruit bats (*Artibeus jamaicensis*) without causing clinical symptoms, and viral shedding occurred for up to 9 days after initial infection [126]. Nonetheless, the majority of human infections are suggested to arise via contact with the intermediate host transmitting the virus, the dromedary camel [127].

Sero-epidemiological studies have shown that dromedary camels in the Arab region had a significantly higher MERS-like CoV seroprevalence compared to other types of livestock [128,129,130,131,132,133]. In addition, a cross-sectional serological study involving over 10,000 individuals across Saudi Arabia found that MERS-CoV seropositivity was 15-fold higher in camel shepherds and 23-fold higher in slaughterhouse employees [134]. Moreover, MERS-CoV has been observed to spread via human-to-human transmission, as evidenced by the high infection rate among members of the same household as well as among healthcare workers in close proximity with infected patients [135,136]. In fact, in an analysis of the medical records of 249 confirmed MERS cases, community-acquired and nosocomial infections were the cause of a substantial proportion of cases [137].

#### 4.1.1. Jordan

The first detected cases of MERS-CoV in Jordan occurred in the governorate of Amman during the 2012 MERS-CoV outbreak, resulting in 2 fatalities out of 12 confirmed cases [138]. At that time, the patients were not rapidly quarantined as there were no established procedures for the identification and tracking of suspected MERS-CoV infections [138]. In contrast, an analysis of 2427 children hospitalized in Amman for acute respiratory illness between 2010 and 2012 did not detect any positive cases of MERS-CoV [139]. In 2013, livestock species encompassing dromedary camels, cows, goats, and sheep were tested for MERS-CoV. The virus was found to be highly prevalent among camels in Jordan and phylogenetically similar to nosocomial strains [129,140]. In 2015, a MERS-CoV outbreak occurred in the governorates of Amman and Zarqa that resulted in 7 fatalities out of 16 laboratory-confirmed cases that were connected to a common transmission chain [141]. The MERS-CoV strain responsible for the 2015 outbreak were more than 99.7% similar to those from Riyadh, Saudi Arabia [142].

#### 4.1.2. Saudi Arabia

Saudi Arabia has been more severely affected by MERS-CoV since its emergence in 2012, over 80% of confirmed cases were from Saudi Arabia [143]. Every year, between 2 and 3 million people from over 180 countries congregate in certain areas of Mecca to perform the Hajj rituals, recreating crowded conditions that have made the transmission of respiratory tract infections a common occurrence among pilgrims [144]. While SARS-CoV and MERS-CoV have never been isolated from Hajj pilgrims, the most commonly isolated viruses were the rhinovirus, influenza virus, and non-MERS coronaviruses, which were associated with severe community-acquired pneumonia in Hajj pilgrims hospitalized in 2013 [145]. However, the impact of MERS-CoV and influenza coinfection on the severity of acute respiratory symptoms is yet to be fully determined [146].

During the Hajj of 2012 and 2014, a response team was dispatched to monitor and gather data on MERS-CoV in cities with major exposure to pilgrims, namely Mecca and Medina, but no positive cases were detected [147]. However, in 2014, a Malaysian pilgrim returning from the Umrah was reported to test positive for MERS-CoV infection and later succumbed to the disease [148]. Additionally, an infected traveler returning to South Korea from Jeddah, Saudi Arabia, was the cause of the largest MERS-CoV outbreak outside of the Middle East, which resulted in more than 30 deaths and billions in socio-economic losses [149]. Among French [150,151,152], Australian [153], Kashmiri [154], Egyptian [155], Chinese [156], Ghanaian [157], Indonesian [158], Iranian [159], and Jordanian [160] pilgrims returning from the Hajj between 2012 and 2016, no MERS-CoV cases were detected, but there was a relatively high incidence of viral pathogens associated with acute respiratory infections. Additionally, a screening of 5235 pilgrims from 22 countries revealed no cases of MERS-CoV during the 2013 Hajj [161]. Contrastingly, two pilgrims returning to the Netherlands from the 2014 Hajj tested positive for MERS-CoV infection [162].

Zoonotic MERS-CoV transmission poses an especially high risk for pilgrims during the Hajj, as each pilgrim is required to sacrifice one sheep, although one camel may be sacrificed for every seven pilgrims. A longitudinal study of permanent workers in one of eight Hajj abattoirs found a low risk of zoonotic infection, but it did not take into account the temporary workers that make up the majority of the Hajj season workforce [163]. Contact with dromedary camels is a particular issue as it was implicated in sporadic MERS-CoV outbreaks reported in 2017 and 2018, indicating that zoonotic transmission can and continuously occurs [164]. Moreover, local camels were shown to have a higher prevalence of MERS-CoV infection compared to camels imported from Sudan and Djibouti [165]. As a result of the MERS-CoV outbreaks, the Saudi Arabian government banned the slaughter of camels during the 2015 Hajj, and it forbade the entry of camels into the holy cities of Medina and Mecca [166].

### 4.2. SARS-CoV-2

The emerging cases of pneumonia of unknown etiology were first identified in the city of Wuhan, China, in the latter months of 2019 and it later became known as COVID-19 [167]. SARS-CoV-2 was later identified as the causal agent. It is currently the cause of an ongoing pandemic that, as of 29 November 2020, has resulted in 63,059,576 confirmed cases and 1,464,849 deaths [116]. The symptoms of COVID-19 most commonly include fever, dry coughing, and fatigue, but it can also involve cardiovascular, gastrointestinal, neurological, and severe respiratory symptoms [168].

The SARS-CoV-2 genome encodes a number of structural, non-structural, and auxiliary proteins, the most important of which is the spike (S) glycoprotein that binds to the angiotensin-converting enzyme 2 (ACE2) host receptor providing entry to the respiratory tract [169]. Compared to SARS-CoV, SARS-CoV-2 has a higher ACE2-binding affinity due to its receptor-binding domain having a more compact conformation and specific residue changes [170]. In fact, efficient human-to-human transmission of SARS-CoV-2 has been attributed to the affinity to the ACE2 host receptor [171].

Close relatives of SARS-CoV-2 have been identified in a metagenomic analysis of samples from *Rhinolophus* species, particularly *R. malayanus*, in Yunnan Province, China [172]. SARS-CoV-2 has been shown to have an 82% shared nucleotide identity with human SARS-CoV and 89% similarity with the bat SARS-like-CoVZXC21 [173]. In another study, the SARS-CoV-2 genome was found to be highly similar to both SARS-CoV (79.6% shared identity) and RaTG13 (96.2% identity), a bat CoV [174]. Investigation of the receptor-binding domains (RBD) of the S glycoproteins and ACE2 host receptors suggests that numerous mammal species may have been capable of acting as intermediate hosts facilitating the transmission of SARS-CoV-2 from bats to humans [174]. However, at this point in time, there is no reliable information on the potential intermediate hosts of SARS-CoV-2, or even if an intermediate host was required [175,176].

#### 4.2.1. Jordan

The first COVID-19 case in Jordan was reported on 3 March 2020, leading to a series of stringent government responses that included a lockdown on all border arrivals, the imposition of a full curfew, and the isolation of administrative governorates from one another [177]. Despite initial successes maintaining a low infection rate, as of 29 November 2020, the number of laboratory-confirmed COVID-19 cases in Jordan has reached 214,307, a figure that translates to 20,918 cases per million and 2694 deaths [116]. The lockdown dealt a severe blow to Jordan’s economic growth, illustrating the need for effective disease surveillance that will prevent such measures from being necessary for the future [178]. Although the lockdown period affected Jordan’s refugee population, a survey of 3362 Syrian refugees in the Zaatari camp did not reveal a statistically significant effect on mental health conditions [179]. Civil society organizations continue to play an important role in ensuring that COVID-19-related awareness and health service delivery is available to vulnerable populations of Jordan’s refugee camps [177,180].

#### 4.2.2. Saudi Arabia

In Saudi Arabia, the first COVID-19 case was confirmed on 2 March 2020, a finding which elicited a swift and decisive government response that included the temporary suspension of the Umrah and other mass gathering events [181]. The reason for this bold measure was the accumulating evidence that crowded places of worship in Iran, Malaysia, South Korea, and Singapore amplified the risk of SARS-CoV-2 transmission [181]. As of 29 November 2020, the number of laboratory-confirmed COVID-19 cases in Saudi Arabia reached 357,128, which is equivalent to 10,193 cases per million and 5884 deaths [116]. Throughout the pandemic, the main concern for Saudi-Arabian policymakers was the Umrah, which was temporarily suspended amid fears that it precipitates the super-spread of COVID-19 among international pilgrims [182]. The decision to suspend the Umrah comes at substantial economic and diplomatic costs to the Saudi Arabian government, which once again underlines the need for apt disease surveillance mechanisms that would prevent such measures from being necessary in the future [183]. 

## 5. Compliance with the International Health Regulations

The International Health Regulations (IHR), first issued in 1959 and substantially revised in 2005, are an international piece of legislation dedicated to preventing and controlling the international spread of infectious disease. Before their revision, the IHR were constrained to a narrow scope of the same three infectious diseases, i.e., cholera, plague, and yellow fever, that were addressed at the 1st International Sanitary Conference in 1851 [184]. The process of modernizing the IHR, which formally began in 1995, was repeatedly delayed due to concerns of its effects on global trade, especially those governed by the World Trade Organization (WTO) agreements [185]. Upon the completion of its revision in 2005, the update IHR maintained its mission of security without unnecessary interference to international traffic and trade, but it shifted the scope of health conditions to cover any “public health emergency of international concern” [184,185]. The updated IHR granted the World Health Organization (WHO) with the authority of making “temporary and standing recommendations for national health measures”, requiring “member states to maintain capacity for surveillance and response”, and allowing the WHO to “access and use non-governmental sources of surveillance information” [184,185].

**Figure 2:**
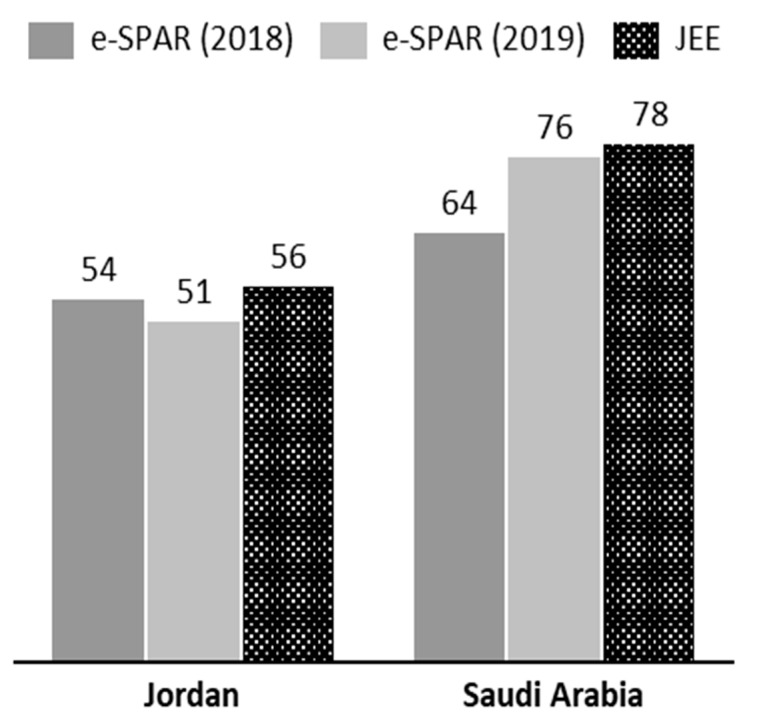
Operational readiness index as reported via the Electronic State Parties Self-Assessment Annual Reporting Tool (e-SPAR) in 2018 and 2019 and as observed by the WHO’s Joint External Evaluation (JEE) mission reports. [Level 1 ≤ 20 (very little capacity), Level 2 ≤ 40 (Little capacity), Level 3 ≤ 60 (Moderate Capacity), Level 4 ≤ 80 (High Capacity), Level 5 < 80 (Well Advanced Capacity)].

Despite these powers, ensuring the compliance of member states with the IHR remains a difficult task for the WHO, and a wide variation in levels of IHR compliance exists between member states [186]. Using the indices developed by Kandel et al. (2020), the operational readiness capacities for Jordan and Saudi Arabia were investigated [186]. As illustrated in Figure 2, there is some discrepancy between the scores given by the WHO in their Joint External Evaluation (JEE) mission reports and those self-reported via the Electronic State Parties Self-Assessment Annual Reporting Tool (e-SPAR). The JEE is carried out by a WHO mission, while the e-SPAR scores are assessed by the country itself. The low self-assessment score could be due to a lack of full understanding of capacity indicators and scores. The JEE mission report was undertaken in 2016 for Jordan (https://www.who.int/ihr/publications/WHO-WHE-CPI-2017.1/en/) and in 2017 for Saudi Arabia (https://www.who.int/ihr/publications/WHO-WHE-CPI-2017.25.report/en/). Saudi Arabia was given a score of 78 by the JEE mission in 2017, but it gave itself scores of 64 in 2018 and 76 in 2019. It is worth noting that the e-SPAR assessments are self-reported and not independently verified by the WHO. Taking a closer look at the capacity indices (Table 2), several of Jordan’s capacities are ranked at 3 due to poor decentralization, with capacities at the governorate and district levels in need of consolidation. In contrast, Saudi Arabia’s capacities are scored at 4, indicating a functional capability at both the national and sub-national levels [186].

One of the most important IHR pillars is disease surveillance, which is defined as the ongoing systematic collection, analysis, and interpretation of data to disseminate relevant findings to key public health stakeholders, as it is essential for the proper functioning of any national healthcare system [187]. To improve disease surveillance capacities, the Field Epidemiology Training Program (FETP), which is hosted by each country’s Ministry of Health (MoH), aims to train an international and interconnected cadre of field epidemiologists to better contain outbreaks before they progress into full-blown epidemics [188]. In terms of the COVID-19 pandemic, the FETPs in the Eastern Mediterranean region, including those in Jordan and Saudi Arabia, actively participated in airport surveillance and public communication efforts [189].

### 5.1. Jordan

In partnership with the WHO, the MoH has implemented a public health surveillance framework that covers over 250 primary and secondary healthcare institutions across Jordan and includes the continuous training of hundreds of health professionals [190]. Correspondingly, a review mission carried out by WHO found that a consolidated Notifiable Disease Surveillance System was the main attribute of Jordan’s health information system [191]. In contrast, a survey of 223 Jordanian physicians in public hospitals found that the majority had not been trained in health surveillance nor filled a report for notifiable diseases, as disease notification was not enforced in Jordanian hospitals [192].

The FETP in Jordan was established in 1998 and is housed within the MoH, where it has trained dozens of physicians in the fields of disease surveillance and outbreak investigation [193]. However, the focus of Jordan FETP surveillance has mostly centered on non-communicable diseases such as diabetes, hypertension, and obesity [194]. As a consequence of implementing the FETP, Jordan is a rarity in the region in that it meets the international standard of one field epidemiologist for every 200,000 people [193]. With regard to the Syrian crisis, the FETP has established a system for reporting the health of Syrian refugees seeking care at public hospitals, thus allowing the MoH to communicate key findings to international organizations every month [193]. In addition, the National Tuberculosis (TB) Program in Jordan detects and treats the disease among Syrian refugee communities, the members of which suffered from a disproportionately higher rate of TB compared to the rest of Jordan’s population [195]. Infectious and parasitic agents were the second most common causes of skin diseases among 799 Syrian refugees profiled as part of an international field-mission assessment [196].

### 5.2. Saudi Arabia

During the annual Hajj, healthcare is provided by the MoH to pilgrims free of charge, although some pilgrims also have access to the physicians accompanying their tour group [197]. As soon as the Hajj season ends, the MoH seeks technical consultations from international public health agencies and begins preparing for next year’s Hajj [198]. In 2012, the Saudi Arabian government established the Global Center for Mass Gathering Medicine to develop its health infrastructure in the context of pilgrimage and enhance research in the emerging field of mass gathering medicine [199]. Like Jordan, Saudi Arabia has also established its own FETP program in 1989 as a joint effort between the MoH and King Saudi University, and it is the only program of its kind in the Gulf Arab states [200,201]. With regard to infectious disease, major health risks to pilgrims include bat-borne diseases such as the coronaviruses [202].

## 6. Conclusions

As frequent hosts of mass gathering and mass influx events, Jordan and Saudi Arabia are presented with significant public health challenges. The ongoing COVID-19 outbreak is evidence that mass gatherings have the potential to substantially amplify the spread of disease, propelling its reach far and wide. In fact, Jordan experienced a surge in COVID-19 cases after a mass gathering during a wedding, in which 21.7% of attendees were infected [203]. As developing countries, Jordan and Saudi Arabia still have much to achieve in terms of their disease surveillance, biosecurity, and biosafety capabilities. Moreover, the presence of bat species that have been associated with dangerous pathogens highlights the ongoing threat of spillover and host switching into human populations. These emerging pathogens often switch hosts by changes in behavior or socioeconomic, environmental, or ecologic characteristics of the hosts. Further investigation is required to determine the level of risk posed by bat-borne viral infections in both countries.

## 7. Recommendations and Future Directions

Actions are needed to prepare for future pandemics, and these actions rely mainly on communities’ preparation and public health measures through the improvement of healthcare, emergency planning, education, and economic systems. Establishing well-designed education and training programs for healthcare workers is key for the implementation of reliable and sustainable practices during outbreaks. Countries need to consolidate their medical stockpiles ahead of any future disease outbreaks to avoid any shortages, which was a common issue in many countries during the early stages of the COVID-19 pandemic. Moreover, ongoing international collaboration is necessary for the development and effective distribution of vaccines. In addition, unified actions on travel restrictions and safety guidelines will also assist in the worldwide control of the disease. Finally, building a strong international collaborative effort is recommended to strengthen global disease surveillance networks, employ and enhance biosecurity management capacity, and promote a One Health concept.

## Figures and Tables

**Table 1:** Bat species native to Jordan and Saudi Arabia along with their worldwide associated pathogens. A dot indicates that this bat species has been recorded in the country.

Bat Species (Common Name)	Jordan	Saudi Arabia	Associated Pathogen
**Emballonuridae**			
*Taphozous nudiventris*	●	●	n/a
*Taphozous perforatus*	●	●	MERS-CoV [67]
**Hipposideridae**			
*Asellia tridens*	●	●	n/a
**Molossidae**			
*Chaerephon pumilus*		●	Chikungunya virus; Dakar bat virus; Entebbe bat virus [10]
*Mops midas*		●	n/a
*Tadarida teniotis*	●		MRV [59]; multi-drug resistant *E. coli* [68]
*Tadarida aegyptiaca*	●		n/a
**Nycteridae**			
*Nycteris thebaica*		●	Duvenhage lyssavirus [69]
**Pteropodidae**			
*Rousettus aegyptiacus*	●	●	EBLV-1 [60]; herpesviruses [70]; Marburg viruses [71,72]
**Rhinolophidae**			
*Rhinolophus blasii*	●		SARS-related CoV [73]
*Rhinolophus clivosus*	●	●	Novel β-CoV [74]
*Rhinolophus euryale*	●		SARS-related CoV [73]
*Rhinolophus ferrumequinum*	●		Adenoviruses [70]; α-CoV [75]; β-CoV [76]; EBLV-1 [77]; γ-herpesvirus [78]; SARS-related CoV [73,75,79]
*Rhinolophus hipposideros*	●	●	β-CoV and SARS-like CoV [80]; MRV [59]
*Rhinolophus mehelyi*	●		SARS-related CoV [73]
**Rhinopomatidae**			
*Rhinopoma hardwickii*	●	●	Hepe-Astroviruses, hepatoviruses, paramyxoviruses & rotaviruses [81]
*Rhinopoma microphyllum*	●	●	n/a
**Vespertilionidae**			
*Eptesicus* *bottae*	●		n/a
*Eptesicus nasutus*		●	n/a
*Eptesicus serotinus*	●		Astroviruses [82]; β-CoV [83]; EBLV-1 [84,85,86]; γ-herpesvirus [87]
*Miniopterus schreibersi*	●		EBLV-1 [77]; Lleida bat lyssavirus [88,89]; Lloviu virus [90]; WCBL [91]
*Myotis capaccinii*	●		EBLV-1 [65]
*Myotis emarginatus*	●		α-CoV & paramyxoviruses [79]; astroviruses [82]
*Myotis nattereri*	●		α-CoV [76]; Bokeloh bat lyssavirus [92,93]; EBLV-1 [77]; herpesviruses [94]
*Nycticeinops schlieffeni*		●	n/a
*Nyctalus noctula*	●		Adenoviruses [70]; astroviruses [82]; β-CoV [95]; hantavirus [96]
*Otonycteris hemprichii*	●	●	n/a
*Pipistrellus ariel*	●		n/a
*Pipistrellus bodenheimeri*	●		n/a
*Pipistrellus kuhlii*	●	●	α-CoV [95]; β-CoV [97]; MRV [59]; novel rhabdovirus [98]; paramyxoviruses [76]; Toscana virus [99]
*Pipistrellus nathusii*	●		EBLV-1 [100]
*Pipistrellus pipistrellus*	●		EBLV-1 [100]
*Pipistrellus rueppellii*	●		n/a
*Plecotus austriacus*	●		n/a

**Table 2:** Joint External Evaluation (JEE) mission report scores across five capacity indices for Jordan and Saudi Arabia.

Index	Jordan (2016)	Saudi Arabia (2017)
Capacity to prevent	**3**	**4**
Capacity to detect	**4**	**4**
Capacity to respond	**3**	**5**
Enabling function	**3**	**4**
Operational index	**3**	**4**

(**3**) Developed Capacity (**4**) Demonstrated Capacity (**5**) Sustainable Capacity.
